# The Statistics of Mental Diseases in Denmark
*"The Statistics of Mental Diseases in Denmark." By Dr. J. R. Hübertz.


**Published:** 1853-07-01

**Authors:** 


### THE STATISTICS OF MENTAL DISEASES IN DENMARK.*

The Danish government has, on four different occasions, caused researches to be made in relation to the idiots and insane of the kingdom.

The first was made in 1830, but as it was directed mainly to ascertain the number of idiots and insane under fifteen years of age, the figure was comparatively low. The second was made in 1840, the third in 1845, and the fourth on the 1st July, 1847. The three last investigations extending to the

* "The Statistics of Mental Diseases in Denmark." By Dr. J. R. Hübertz.

entire population, and taking cognizance of all cases of disordered mind and alienation, yielded a much larger figure. Thus the first census, that of 1830, gave about 1000 idiots and insane persons, whilst the census of 1847 carried the number to near 4000. It is this last census which will be more particularly examined.

In order to conform to the order of the minister, it was at first sought to adopt the principles of distinguishing cretins and idiots on the one hand, and the insane on the other. The execution of this proved difficult; it became necessary to comprise within the class of idiots, not only all subjects reported as having become born without intelligence, but also those who had become affected at an indeterminate epoch of childhood. The insane are those designated in the returns as having been born with intelligence, which they had subsequently lost. This is the reason why children even of tender age, are found in the class of the insane.

Cretinism and idiotism being, in our opinion, varieties of the same affection, it is scarcely necessary to add that cretins and idiots are comprised under the common denomination of idiots. But it is quite possible that many subjects are classed amongst the idiots, who were nevertheless born with intelligence, and who have been placed amongst the insane.

The returns embrace the territory of Denmark only. Denmark is divided as to its civil administration, into 20 *amts* or prefectures, and about 146 *herreds* or arrondissements. With reference to the administration of matters connected with mental alienation, the plan of making these great divisions of the *amts* and *herreds* has been followed. The first division contains Seeland, and Laland-Falster; the second, Finland; and the third, Jutland.

*The number and relative proportions of idiots and insane.*—According to the census of 1847, there were in Denmark 3756 persons who were more or less deprived of normal intelligence. Of this number, 1865 belonged to the male sex; 1891 to the female, or 49·7 per cent. of males, and 50·3 per cent. of females. (The proportion of the sexes in the entire population at this time was 49·44 per cent. for the males, 50·66 for the females.) This proportion corresponds closely with those found in 1840 and 1845.

The sum total of 3756 is divided into 1995, or 53·12 per cent. idiots, and 1761 or 46·88 per cent. insane. Among the idiots there were 1066 or 53·43 per cent. males, and 929 or 46·47 per cent. females. Among the insane, the proportion of the male sex was 799, or 45·37 per cent.; that of the female sex was 962, or 54·63 per cent.

Out of the idiots 295 were inhabitants of towns (scarcely 11 per cent.) of which 150 were males and 145 females; 1700, or nearly 89 per cent. were inhabitants of the country. Of these last, there were 916 males and 784 females.

The insane were distributed as follows:—in the towns there were 658, or 37 per cent; of these 289, or 43 per cent. were males, and 369, or 57 per cent. females. In the country there were 1103 insane persons, or 63 per cent.; of these 510 or 46 per cent. were males, and 593, or 54 per cent. females.

The following proportions of *idiots* and *insane*, in 1000 inhabitants, were ascertained:—For the whole of Denmark, 2·71 males, and 2·69 females; both sexes, 2·70. In the towns, 3·14 males, and 3·54 females; both sexes, 3·34. In the country 2·60 males, and 2·47 females; both sexes, 2·53.

The following were the proportions of the *insane* in 1000 inhabitants:—In all Denmark, 1·15 males, and 1·35 females. In the towns 2·07 males, and 2·55 females; in the country, 0·92 males, and 1·04 females.

*Of the religion of the persons affected.*—The proportion of idiots and insane in 1000 inhabitants of different sects presented considerable variety. The Catholics showed 3·34 in 1000, the Jews 5·85, and the Calvinists 9·16. But we are especially warned that the data upon this head are not trustworthy.

*Complications.*—The particulars are imperfect. It is, however, observed, that in Denmark goître is very rare; in countries where goître is endemic, there was a disposition to confound it with cretinism, but the Sardinian commission having shown that one-third only of the cretins were affected with goître, this opinion is giving way.

*Causes.*—The returns contain but slender reference to general causes. Dr. Hübertz, however, enters into some interesting inquiries into the influence of the soil, &c., in the production of insanity.

The fertile soil of clay and flints, enriched by detached fragments of chalk from the strata which are found in all the Danish isles, and on the eastern coast of Jutland, gives rise to a population much more dense than the other geological formations. Thus in the islands of Seeland, Finland, &c., there are found from 2500 to more than 4000 inhabitants to the square mile (Danish); in Jutland from 700 to near 3000. Among the dense population of the isles, idiots and insane are found in the proportion of 2 to 3 in 1000; amongst the scattered population of the lignite formation of the west of Jutland, the proportion is 3 to 5 in 1000. We may therefore conclude that the sterility of the soil, and the thinness of the population, must be reckoned among the general causes of the disease.

Another quality, the cohesion of the particles of the soil, must also be taken into consideration. In clayey soils we find the proportion of patients sometimes greater, sometimes more restricted; but in general the number is not excessive. In sandy soils we always find mean proportions. Two districts claim our attention in this respect: the island of Laland in the Baltic, and the arrondissement of Vandfuld, on the north-west coast of Jutland. The soil of both consists equally of clay, either on the surface or in the strata. The only difference is, that the island is flat, whilst the arrondissement has its surface cut up into hills and valleys. Amongst the inhabitants of the island, the smallest proportion of insane has always been found; it seldom rises to 2 or 1000. In the arrondissement the very highest proportion has been found 6 in 1000. But on the other hand, the inhabitants of the island are afflicted to a greater extent with intermittent and remittent fever. The geological conditions being nearly the same, the different morbid circumstances of the two districts must be attributed to other causes, no doubt greatly to climate.

*Climate.*—The largest proportion of insane has been mostly found on the north and north-west frontiers. Again, if we start from the point where the proportion is the smallest, the increase rises pretty regularly by degrees towards the region described, until it reaches the maximum in the north and north-west.

We find the densest groups and the highest proportions in the arrondissements exposed to the north and west, and on the other hand, we find the most scattered groups and the most slender proportions in those which are sheltered from the north and west winds by hills. Thus, to pass from the arrondissement which contains the lowest proportion, into that which contains the highest, we have only to cross the hills which separate them.

If the localities open to the south are those which least favour the development of insanity, they must also be those which have the greatest influence upon its disappearance. It is, therefore, in these localities, that institutions for the insane should be located.

*Age.*—As the disease does not manifest itself in the first days of existence, or is not susceptible of being recognised at this period, the frequency of insanity or idiotcy in the early periods, as ascertained, is low; but a very sensible augmentation will be observed in the proportion of idiots who have reached the age of from 10 to 15 years. The culminating period for male idiots is from 20 to 25 years; that of female idiots from 15 to 20.

For the insane males the culminating period is from 35 to 40, for females, from 45 to 50.

At the time of the census of 1845, it was ascertained that there were 8·38 per cent. of the inhabitants above 60 years of age. In 1845 there were but 4·74 per cent. of idiots above that age, and in 1847, only 3·9 per cent. It may therefore be concluded, that in madness idiotism exercises a great influence in shortening life.

*Civil condition and profession.*—The Danish nation is agricultural; 75 per cent. lives in the country, and 25 per cent. in the towns. The following particulars may be cited. Out of 1000 patients, the parents of 46·37 were ecclesiastics, physicians, teachers, &c., 14·59 were traders; 48·31 artisans, 4·87 soldiers, 2·59 artists, 20·10 vendors of provisions, 830·74 agriculturists, 18·81 labourers, 13·62 natural children, not recognised by any parents.

*The duration of mental alienation.*—The facts in reference to this question, preclude exact conclusions. The mean duration appears to be 13·39 years.

The annual increase of cases is estimated at 135.

*Nationality.*—Dr. Hübertz quotes what is generally reported, concerning the prevalence of insanity in various countries. It cannot escape attention, however, that the data upon this subject, especially for countries beyond the limits of Europe, scarcely justify positive conclusions. We pass over this part of his observations, and cite such of his deductions as refer to Denmark.

The population of Denmark is, for the most part, of the Gotho-Germanic race. In the Baltic islands, however, and especially in the south of Bornholm, in Laland-Falster, the south of Seeland, well-marked traces of Sclaves are found, perhaps the descendants of the colonists of the 13th century; and it is very remarkable that the least elevated proportion of insane is observed in this part of the population.

*Education.*—In Denmark, idiots and insane persons are more numerous in the provinces most removed from the centre of civilization, where education is most neglected, on account of the sterility of the land, and the poverty of the inhabitants. This agrees with what is observed in other countries.

*Marriage.*—Nothing very definite is recorded.

*Abuse of spirituous liquors.*—Since the last twenty or thirty years, the increase of prosperity has brought with it habits of great sobriety. Previously to that period, the most vicious habits of intoxication prevailed.

*As to the personal situation of the patients.*—There were 318 males and 304 females, confined in asylums and hospices; in hospitals, 5 males and 5 females; in poor-houses, 88 males and 115 women; as private boarders, 51 males and 71 females; in prisons, 6 males and 1 female; attended by special domestics, 5 males and 11 females; bound and partly locked-up during paroxysms, 20 males and 12 females; confined in private houses, 75 males and 76 females.

In addition to the idiots and insane which came within the census of 1847, there were other persons who were often subject to periodical attacks of alienation, but who, at that particular time, were exempt. Those persons amounted to 196 males and 194 females.

The analysis we have given of the valuable paper of Dr. Hübertz comprises the points of leading and general interest, with reference to the statistics of mental alienation. The paper contains a number of tables and details concerning the distribution of the insane in the different towns and districts, and other particulars which are chiefly of local interest.

